# Impact of Tumor and Immunological Heterogeneity on the Anti-Cancer Immune Response

**DOI:** 10.3390/cancers11091217

**Published:** 2019-08-21

**Authors:** Carolyn Shembrey, Nicholas D. Huntington, Frédéric Hollande

**Affiliations:** 1 Department of Clinical Pathology, Victorian Comprehensive Cancer Centre, The University of Melbourne, Melbourne, VIC 3000, Australia; 2 Centre for Cancer Research, The University of Melbourne, Melbourne, VIC 3000, Australia; 3 Department of Biochemistry and Molecular Biology, Biomedicine Discovery Institute, Monash University, Clayton, VIC 3800, Australia

**Keywords:** tumor heterogeneity, natural killer cells, tumor mutation burden, immunotherapy

## Abstract

Metastatic tumors are the primary cause of cancer-related mortality. In recent years, interest in the immunologic control of malignancy has helped establish escape from immunosurveillance as a critical requirement for incipient metastases. Our improved understanding of the immune system’s interactions with cancer cells has led to major therapeutic advances but has also unraveled a previously unsuspected level of complexity. This review will discuss the vast spatial and functional heterogeneity in the tumor-infiltrating immune system, with particular focus on natural killer (NK) cells, as well as the impact of tumor cell-specific factors, such as secretome composition, receptor–ligand repertoire, and neoantigen diversity, which can further drive immunological heterogeneity. We emphasize how tumor and immunological heterogeneity may undermine the efficacy of T-cell directed immunotherapies and explore the potential of NK cells to be harnessed to circumvent these limitations.

## 1. Introduction

Recent advances in our understanding of cancer, driven by the development of sophisticated biochemical and molecular techniques, have highlighted the complex and heterogenous nature of this disease. Within individual tumors, significant differences in the molecular and phenotypic profiles may arise from tumor cell-intrinsic or extrinsic factors. Genomics has provided the most extensive insights to date about tumor-intrinsic variations, with sequencing studies revealing a large extent of clinically-relevant intra-tumor heterogeneity [1,2,3]. Thus, next generation sequencing of multiple tumor types identifying the association between increased clonal heterogeneity and higher pathological stage and/or worse prognosis [4]. Moreover, genetic heterogeneity has also been identified across patients, and the incidence of clinically actionable mutations differs significantly between tumors arising from different tissue or cell types, amongst patients with the same class of tumor, and between matched primary and metastatic tumors within the same patient [5,6,7,8]. Non-genetic determinants of heterogeneity have also garnered significant interest, as even genetically identical cells may harbor unique chemosensitivity profiles [9]. This points towards the role epigenetic modifications [10,11,12] and metabolic reprogramming [13], in dictating the functional variation observed within individual populations.

Tumor cell extrinsic factors, such as the cellular and structural elements of the tumor microenvironment (TME), are also known to influence tumor heterogeneity. For instance, the spatial arrangement of cells with receptor tyrosine kinase amplifications in glioblastoma has been shown to correspond with degree of vascularization in the local TME [14]. Similarly, in melanoma patients, the extent of subclonal divergence from the mutational profile of the primary tumor is dependent on the metastatic site [15], suggesting an influence of the local microenvironment. Perhaps the most important component of the TME are the immune cells. Whilst the tumor-sculpting role of the anti-cancer immune response has long been recognised, conflicting reports exist on the impact of this immunoediting on tumor heterogeneity. The selective pressure of the immune response has been shown to profoundly reduce sub-clonal diversity via the targeted elimination of immunogenic cell variants [16], recent evidence indicates that the adaptive immune response may in fact potentiate genomic instability [17], thus promoting the rise of novel subclones thereby increasing tumor heterogeneity. As heterogeneity within the immune or tumor cell compartments could conceivably impact the efficacy of immunotherapies, there is a vital need to improve our understanding of the relationship between the two.

## 2. Spatial, Functional, and Temporal Heterogeneity of Immune Cell Infiltrates

Tumor cells develop in a dynamic niche; individual tumor cell subpopulations not only compete and cooperate with each other, but also with the surrounding TME and its constituent immune cells. Single-cell sequencing studies have confirmed that tumors may be populated by a vast and diverse array of immune components: innate leukocytes, such as natural killer (NK) cells and mast cells; phagocytes, such as macrophages, neutrophils, and dendritic cells; and adaptive effectors, including naïve, memory, and effector B- and T-lymphocytes [18]. It is clear that the degree of immune infiltration and the composition of this infiltrate can vary markedly across tumor types [19] and stages [18], as well as between patients with the same tumor type [20,21]. Similarly, whether synchronous metastases within a single patient regress or progress has been associated with their distinct immune profile [22]. Within a tumor, complexity is further compounded by the differing spatial distribution of immune effectors between the core and invasive fronts, as well as within the adjacent tertiary lymphoid structures [23,24].

However, as increased infiltration of CD8+ T-cells is prognostic for better outcome in numerous tumor types [25,26,27,28], traditional scoring of tumor immunogenicity has been based upon the degree of T-cell inflammation alone. Immunologically ’hot’ tumors, such as melanoma and non-small cell lung cancer (NSCLC), present with a high degree of T-cell permeation, whereas tumor-infiltrating lymphocytes (TILs) are scarcely observed in ’cold’ tumors, such as ovarian, prostate, and pancreatic cancers. More recently, a third immunologically ’altered’ phenotype has been proposed, denoting cases where peri-tumoral sites are densely inflamed with immune cells which lack the ability to infiltrate into the tumor [29]. As the T-cell inflamed gene expression profiles of ’hot’ tumors have been strongly linked with positive response to checkpoint blockade therapies [30,31], significant attention has been focused on developing therapeutic strategies which can convert immunologically ’cold’ or ’altered’ phenotypes into ’hot’ environments [32,33,34]. Yet, as the immune contexture may vary across non-adjacent tumor regions, it should be emphasized that many tumors may not be universally ’hot’ nor ’cold’.

To address this disparity, the Immunoscore method proposed by the Galon group incorporates spatial context into its immunological quantitation metric, computed by the ratio of memory CD3+ and cytotoxic CD8+ TILs at the tumor centre and invasive margins. In colorectal cancer (CRC), this index has been validated as an independent prognostic marker which performs better than both Tumor-Node-Metastasis (TNM) staging and microsatellite instability (MSI) status [35,36,37] and an in vitro diagnostic assay has been made clinically available for assessing relapse risk in Stage II and III CRC. Yet, there is substantial evidence that quantification varies between non-adjacent areas of tumor biopsies [38,39,40], suggesting that single biopsies may not be representative of the broader infiltrating immune landscape. Pertinently, a phenomenon termed Immunoskew has been documented, whereby a minority of tumor regions are densely infiltrated with TILs despite an otherwise barren TME [41]. Determining whether Immunoskew extends to other immune cell populations, and identifying the specific intra-tumor differences which drive this differential infiltration pattern, are worthy areas for future study.

Beyond TILs, the contribution of other cell types to tumor immunogenicity should not be overlooked. NK cells are inversely correlated with cancer incidence [42,43] and intra-tumoral NK cell infiltrates have been identified as a positive prognostic marker in multiple solid cancers [44,45,46,47,48] and haematological malignancies [49]. Additionally, NK cells are supremely important in the control of metastasis. A wealth of in vivo studies have demonstrated that mice depleted of NK cells via pharmacological inhibition [50,51,52] or genetic knockout [53] are more vulnerable to metastasis than their NK cell-proficient counterparts. The same is true for mice reconstituted with NK cells deficient in cytotoxic molecules, such as perforin and interferon-γ (IFNγ) [54,55] or activating receptors [56,57,58,59]. This notion has been confirmed in a clinical study of CRC liver metastases, where increased frequency of intra-tumoral NK cells was the variable most significantly (*p* = 0.01) associated with better overall survival, performing better than other clinical parameters including TNM stage, number/size of metastases, and frequency of infiltrating CD3+ lymphocytes [60]. Additionally, there is strong evidence supporting the role of NK cells in the clearance of putative cancer stem cells [61,62,63,64], suggesting that NKs may promote long-term recurrence-free survival.

The discordance in immune infiltrate between primary and metastatic tumors is more pronounced in metachronous than synchronous tumors [24,65,66] indicating that temporal changes also contribute to tumor heterogeneity. The composition of immune infiltrates is also known to change as tumors progress, with one study in CRC reporting an increased prevalence of innate immune cells and decreased number of most T-cell lineages in more advanced tumor stages [18]. The latter may be particularly important when considering the age-associated decline in lymphocyte number and function [67], particularly given that the majority of new cancer diagnoses are made in those over the age of 65.

Above all, a limitation of current techniques that quantify immune infiltrates is that they frequently do not assess functionality. Recent evidence suggests that infiltration alone may not be sufficient to elicit anti-tumor responses, as effector cells can be relegated to the peritumoral stroma and therefore lack the direct cell contact required for target cell destruction [68,69,70]. Similarly, the efficacy of each immune cell population may be influenced by the immunoregulatory cytokines produced by neighboring cell types. For example, infiltrating cytotoxic lymphocytes may be restrained by various immunosuppressive cell types, including myeloid-derived suppressor cells [71], Tregs [33,72,73,74], and tumor-associated (TA) fibroblasts [68,75,76,77], which are diversely distributed across cancer types. Conversely, traditionally immunosuppressive cells can act beneficially depending on the surrounding tumor context [78,79].

## 3. Tumor Cell-Driven Immunological Heterogeneity

**Figure 1:**
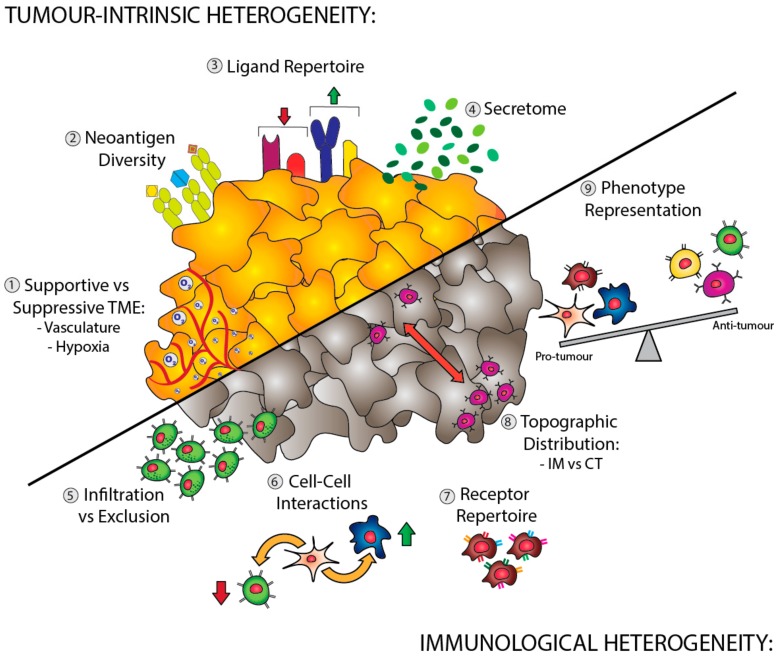
Tumor and immunological heterogeneity. Tumor-intrinsic drivers of heterogeneity (upper left) include diversity in: the degree of tumor vascularization or hypoxia (**1**), which determines whether the local tumor microenvironment (TME) will support or suppress anti-tumor immune cells; the variable expression of neoantigens (**2**) and ligands (**3**), which facilitate interaction with various immune cell types; and the secretion of soluble factors (**4**) (which may also be produced by the immune cells themselves) that may promote or restrain the action of nearby immune cells. Immune cell contributions to heterogeneity (bottom right) include: the type and density of infiltrating versus excluded immune cells (**5**); modulatory interactions between co-localised immune cell types (**6**); the balance of activating versus inhibitory receptors (**7**); effector cell distribution between the invasive margin (IM) and central tumor (CT) (**8**); and the overall balance between pro- and anti-tumor effectors (**9**).

The observation that increased TIL fractions have positive prognostic value in numerous tumor types has culminated in the harnessing of this subset for immunotherapy, primarily in the form of immune checkpoint inhibitors. Whilst strikingly effective in tumor types, such as melanoma, renal cell carcinoma (RCC), and NSCLC, the efficacy of immune checkpoint therapies is highly variable across solid malignancies. For example, in CRC, positive therapeutic responses to T-cell directed checkpoint inhibitors are limited to approximately 30% of patients with MSI, which represents 5% of all patients [80]. Whilst the exact molecular mechanisms which underpin this resistance remain elusive, emerging evidence suggests that broad spectrum of clinical responses could be partially attributable to immunological heterogeneity. As well as differences in immune infiltration and interaction of immune cell types, there are multiple tumor cell intrinsic factors, such as the secretome, receptor–ligand profile, and neoantigen repertoire, which can drive immunological heterogeneity (Figure 1).

### 3.1. Secretome Heterogeneity

Infiltrating immune cells can be conditioned by the soluble factors secreted by nearby tumor cells. Tumor cells can directly foster an immunosuppressive TME via the production of enzymes and metabolites including indolamine 2, 3-dioxygenase (IDO) [81,82], lactic acid [83] and prostaglandin E_2_ [68,84]. As metabolically heterogeneous regions are detectable within discrete tumors [13], it is conceivable that these immunosuppressive metabolites may be irregularly distributed. Although such mediators are directly implicated in the dampening of T- and NK cell activity, their immunomodulatory effects are not reflected in routine clinical immunohistochemistry, where the focus is on assessing the presence or absence of lymphocytes, not their activation state.

There are multiple reports of tumor-derived cytokines, such as transforming growth factor-β1 (TGF-β1) suppressing cytotoxic effector functions [85,86,87], frequently acting via the downregulation of activating receptors [88,89,90]. As TGF-β1 production is exacerbated in hypoxic conditions, it follows that hypoxic tumor cells show heightened resistance to NK cell-mediated killing [91,92,93]. In response to hypoxia, accumulation of immunosuppressive adenosine and subsequent signaling via the A2A adenosine receptor has been shown to potently inhibit T- and NK cells [94,95]. This tumor-protective effect is abrogated in hyperoxic conditions [96,97], suggesting that supplemental oxygen could be a useful co-adjuvant for immunotherapy. Due to the disorganized vascularization of growing tumors, tumor cells may be irregularly exposed to hypoxia [98]. Interestingly, this intermittent hypoxic conditioning has been shown to enhance inflammatory responses as compared with chronic hypoxia [99,100,101]. However, this phenomenon has also been shown to enhance tumor growth and promote radiotherapy resistance in in vitro and in vivo models [102]. Thus, more research interrogating the role of intermittent hypoxia in the context of the TME would be valuable.

Additionally, there is mounting evidence that different immune cell subtypes, particularly NK cells, may exhibit tropisms for different tumor types. Human NK cells develop from CD34^+^ hematopoietic progenitors in the bone marrow and critically rely on interleukin-15 (IL-15) transpresentation for maturation into two functionally distinct mature NK cell subsets in the periphery [103,104], divided based on CD56 expression. Approximately 90% of circulating NK cells exhibit the CD56^dim^ phenotype, which primarily function as cytolytic effectors via production of perforin and granzyme B. Conversely, the immunoregulatory CD56^bright^ subset is charged with production of type I pro-inflammatory cytokines (IFNγ, Tumor necrosis factor (TNF)α, GM-CSF, IL-10, IL-13) and preferentially reside in the secondary lymphoid organs. In breast cancer [105] and gastrointestinal stromal tumours (GIST) [44], tumor-infiltrating NK cells are primarily of the poorly cytotoxic CD56^bright^ subtype, whereas glioblastomas are preferentially infiltrated by CD56^dim^ NK cells [106], and conflicting tropisms have been reported in NSCLC [107,108]. Such differences in NK cell homing may also be associated with the extent of hypoxia in the TME, as hypoxia-induced upregulation of chemokines C-X-C chemokine receptor type 4 (CXCR4) and CCR7 has been shown to favor migration of the CD56^bright^ subset [109]. Intriguingly, this is unlikely to be explained by chemokine profile alone, as NK cell infiltration in CRC is scarce despite elevated expression of chemokines that attract CD56^bright^ (CXCL9, CXCL10, CCL3, CCL4) and CD56^dim^ (CXCL8, CXCL1, CXCL5, and CXCL12) subsets in tumor tissue as compared with adjacent normal mucosa [69].

### 3.2. Receptor–Ligand Heterogeneity

Through somatic recombination, the adaptive immune system is able to generate immunoglobulin and T-cell receptor (TCR) repertoires which span millions of antigens. Disparate receptor repertoires also exist within the NK cell compartment and underpin their functional heterogeneity. NK cell effector functions are tightly controlled by a complex network of activating and inhibitory receptors, and the ability of NK cells to eliminate target cells and produce cytokines relies upon the integration of signals from both types. Activating receptors, such as the natural cytotoxicity receptors (NKp30, NKp44, and NKp46) and NKG2D, recognise stress-induced ligands which are upregulated in response to DNA damage or viral transformation (“induced-self” recognition) [110,111]. Conversely, inhibitory receptors comprising the highly polymorphic killer cell immunoglobulin-like receptor (KIR) family work to prevent the aberrant targeting of healthy host cells by engaging “self” molecules, such as major histocompatibility complex class I (MHC-I), glycoproteins, and cadherins, and accordingly targeting those that have lost expression of these molecules (“missing-self” recognition).

Whilst NK cell receptors are preformed and, therefore, do not undergo the rearrangements characteristic of B- and T-cell receptors, a remarkable degree of NK cell diversity is conferred by the combinatorial expression of different NK receptors. Utilising a mass cytometry panel of 28 NK cell receptors, Horowitz et al. successfully detected up to 30,000 distinct NK cell phenotypes within a healthy individual. Such heterogeneity may in part be explained by the multiple factors which can regulate NK cell receptor repertoires, including host-genetics [112,113], epigenetic regulation [114] and previous viral infection [115,116,117].

The KIRs are the most heterogeneously expressed family of receptors. KIRs are encoded by 15 highly polymorphic genes clustered in the leukocyte receptor complex on chromosome 19q13.4 [118]. CD56^dim^ NK cells express between 7 and 11 KIR family members; the presence or absence of individual KIR genes in each haplotype generates considerable genotypic diversity, which is compounded by differing allelic frequencies within each gene. Such heterogeneity is of clinical importance, as KIR-mismatch is a prerequisite for the graft-versus-leukaemia effect of allogenic NK cell transfer [119,120,121] and specific KIR genotypes have been associated with better responses to combination immunotherapies in neuroblastoma patients [122,123]. Similarly, three splice variants of the activating receptor NKp30 have been identified and the relative abundance of activating versus inhibitory isoforms has been associated with clinical outcome in neuroblastoma [124] and gastrointestinal sarcoma [44]. In the latter study, expression of inhibitory NKp30c as the most abundant isoform was the only independent prognostic factor for overall survival, whose overexpression was traced to a single nucleotide polymorphism in the natural cytotoxicity receptor-3 (NCR3) gene [44].

Importantly, numerous in vitro studies have demonstrated the ability of tumor cell lines to differentially regulate the receptor repertoires of NK cells [62,63,125]. Coordinated patterns of receptor dysregulation have similarly been documented in tumor-infiltrating as compared with peripheral NK cells. Reduced expression of activating receptors (including NKp30, NKp46, NKp80, CD16, DNAX accessory molecule-1 (DNAM-1) and NKG2D) has been documented in lung carcinoma [108], breast cancer [105] and acute myeloid leukaemia [126]. In each case, functional analysis of these patient-derived NK cells revealed that tumor-associated NK cells are poor producers of IFNγ and have an impaired ability to degranulate, although these studies did not investigate whether these defects impacted clinical outcome. Conversely, upregulation of the CD96/NKG2A inhibitory receptor complex has been observed in renal cell [127] and associated with poor prognosis in hepatocellular [128] carcinomas.

Immune cell responsiveness is not only determined by the balance of receptors present on a given cell, but also by the various ligands expressed by the target cell. For instance, a recognised mechanism of tumor escape in is the shedding of soluble ’decoy’ ligands for NK cell activating receptors, including BCL2-associated athanogene 6 (BAG-6) [129,130] and B7-H6 [131]. Interestingly, a genome-wide knockout screen performed by Klein and colleagues [132] identified loss of B7-H6 as the sole event which increased resistance of the chronic myeloid leukaemia cells to NK cell killing. Yet, recent studies investigating the functional consequences of NKG2D ligand shedding have challenged the idea that soluble ligands are exclusively immunosuppressive; in human cancers, shedding of MHC class I polypeptide related sequence A (MIC-A), a low-affinity NKG2D ligand, facilitates immune evasion [133,134]; however, shedding of the high-affinity murine analogue, MULT-1, enhances NK cell activation and tumor rejection [135].

Another major mechanism by which tumors evade immune destruction is up-regulation of immune checkpoint ligands, such as CD80/86, 4-1BBL, and OX40-L. Immune checkpoints are a broad group of inhibitory pathways and co-receptors with the primary purpose to restrict the duration and amplitude of an immune response, thereby minimizing collateral damage to healthy tissues [136]. Immune checkpoints primarily regulate T-cell responses, although checkpoint expression has been documented in B cells, NK cells and professional antigen-presenting cells (APCs) [136]. In the context of cancer, chronic antigen exposure coupled with engagement of inhibitory immune checkpoint ligands on tumor cells results in effector T-cell exhaustion, wherein T-cells undergo profound impairment of proliferation, cytokine production and cytotoxicity. Even in hostile immune environments densely infiltrated with cytotoxic T-lymphocytes, checkpoint ligand expression impinges upon tumor clearance [137]. Programmed Death Ligand 1 (PD-L1) has attracted particular attention in that its expression is associated with poor prognosis in multiple cancers [138,139,140,141]. Indeed, six of the seven FDA-approved immune checkpoint inhibitors target the PD-1/PD-L1 inhibitory axis [142]. PD-L1 expression by tumor cells is a strong predictive biomarker for response to PD-L1 blockade [143], although positive therapeutic responses to have been reported PD-L1-knockout mice [144] PD-L1-negative patients [145]. This suggests that whilst PD-L1 positivity enriches for responders, combining PD-L1 expression with other predictive factors, such as MSI status, may increase our confidence in patient selection. Indeed, even in tumors classed as PD-L1-positive, individual tumor cells vary widely in terms of PD-L1 expression [66,146]. Individual research groups set thresholds for ligand positivity ranging from 1–50% [147] and in tumors classed as checkpoint-positive, negative-staining cells may be ignored during clinical decision making despite their likely influence on treatment efficacy. Likewise, ligand profiles are labile in response to therapy; conventional chemotherapeutics increase expression ligands for the NK cell activating receptors NKG2D and DNAM-1 in multiple myeloma [148] and ovarian cancer [149] cells.

There is also some degree of binding promiscuity involved in receptor–ligand interactions. An array of NK cell receptors with opposing functional roles compete for binding of CD155 (PVR) ligand, including activating DNAX accessory molecule-1 (DNAM-1) and inhibitory T-cell immunoreceptor with Ig and ITIM domains (TIGIT) [150]. CD96-CD155 ligation is primarily considered an inhibitory checkpoint in the NK-mediated control of metastasis [151], however an activating role for CD96 has also been reported via promoting target adhesion [152]. Such complexity demonstrates how the interplay between immune cell receptors and ligands should be assessed as a network rather than at the single molecule level, and how such assessment should take into account spatial heterogeneity rather than focus on limited areas.

### 3.3. Neoantigenic Heterogeneity; A Challenge for T-cell Directed Immunotherapies

Just as the ability of the immune system to recognise and destroy invading pathogens or foreign particles relies on the ability to distinguish self from non- or altered-self, the genetic marks carried by tumor cells provide a diverse set of antigens that the immune system can use to detect malignant cells amongst their normal counterparts. Accordingly, T-cell directed immunotherapies have currently proven most efficacious in cancer types with high average tumor mutation burden (TMB) [153,154]. Whilst clinical responses to immune checkpoint blockade in cancer types with traditionally low TMB have been reported, these are generally restricted to virally-induced cancers, such as Merkel cell carcinoma and human papilloma virus-positive head and neck squamous cell carcinoma (HPV+ HNSCC), which show enhanced T-cell infiltration due to the presence of viral antigen [155,156]. Similarly, MSI has been identified as a pan-cancer predictive marker for checkpoint inhibitors [157,158], as MSI tumors harbour DNA mismatch-repair defects and thus present with 10–100 fold greater TMB than genomically stable tumors [159]. MSI tumors also have higher TIL density as compared with their microsatellite stable (MSS) counterparts, due primarily to their increased frequency of mutated neo-epitopes recognisable as non-self [160]. Neoepitope load is predictive of clinical outcome in bladder cancer [161], multiple myeloma [162], melanoma [163], and ovarian cancer [163,164], and there several reports of cytotoxic T-cells recognising epitopes derived from single point mutations [165,166,167]. Accordingly, heightened TMB is associated with more diversified expansion of T-cells [168] and greater infiltration of neoantigen-specific clonotypes [169].

Neoepitope targeting is an appealing therapeutic avenue in that the lack of neoepitope expression in healthy cells ensures that neoepitope-specific T-cells are not impinged by central tolerance, thereby conferring greater specificity and less toxicity. To this end, multiple studies are currently investigating the possibility of targeting neoepitopes with for personalised immunotherapy (see Türeci et al. [170] 2016 for a complete list of completed and ongoing trials). Yet, a barrier to the clinical applicability of these strategies inheres in the tremendously diverse range of antigenome landscapes observed between patients. In a recent pan-cancer analysis where almost one million unique neoantigens were identified, only 24 were conserved in at least 5% of patients in one or more cancer types [171]. Similar results have been reported in analyses of individual cancer types [172,173]; of note, a cohort study from The Cancer Genome Atlas (TCGA) in CRC (*n* = 598) revealed that only 4% of predicted neoepitopes were shared by at least two patients [174]. This complexity is compounded by the substantial diversity across patients with respect to human leukocyte antigen (HLA) haplotypes required for antigen presentation. This may be particularly important as, unlike membrane-associated checkpoint molecules, the majority of tumorigenic mutations affect genes which encode for intracellular proteins [175] and are therefore only recognizable by CD8+ T-cells following antigen processing and presentation in the context of MHC-I.

There is also strong evidence supporting the existence of neoantigenic heterogeneity within individual tumors. In lung adenocarcinoma, post-surgical recurrence has been associated with an increased proportion of branched neoantigens, defined as those not homogenously detected throughout the tumor [21]. Importantly, TCR sequencing of 45 tumor regions in these patients demonstrated that the majority of T-cell clones were topographically restricted, and that intra-tumor heterogeneity in TCR repertoires positively correlated with predicted neoantigen variety. Together, these findings suggest that regional differences in T-cell infiltration may be driven by spatially distinct neoantigen profiles, which may have important consequences for the development of therapies which target single neoantigens. There is also accumulating data suggesting that neoantigens are not equally ’potent’ in their ability to elicit T-cell effector functions, highlighting that assessing neoantigen quality may be more important than their quantity. Recent work has demonstrated that qualitative neoantigen prediction models, where fitness is conferred by a higher probability of TCR-recognition, have surpassed quantitative models in their ability to stratify for survival [176,177].

## 4. Neoantigen-Independent Strategies for Immunotherapy

Evidently, neoantigenic heterogeneity presents a formidable challenge in the development of T-cell based immunotherapies. To circumvent this striking degree of variability, clinical attention has been directed towards targeting non-mutated antigens that show heightened tumor specificity, including cancer germline antigens (CGAs). Unlike patient-specific neoepitopes, non-mutated antigens arise from comparatively well-defined mechanisms and are thus more likely to be conserved across patients. CG antigens are proteins that are exclusively expressed by germ cells which can be aberrantly re-expressed in multiple cancers, including the archetypal melanoma antigen (MAGE), synovial sarcoma X-chromosome breakpoint (SSX), and oesophageal squamous cell carcinoma (ESO) families. Expression of CG antigens is epigenetically modulated, being frequently induced following hypomethylation of CpG islands and covalent histone modifications [178]. Due to their absence on healthy somatic cells, CGAs have garnered substantial interest as therapeutic targets. However, development of CGA-directed therapies has been hampered by their low prevalence. Indeed, Kerkar et al. [179] report that only 2–3% of common epithelial cancers uniformly express New York-ESO-1 (NY-ESO-1).

An alternate strategy has been to target TA antigens that, despite basal expression in healthy cells, are preferentially expressed by transformed cells. One class of TA antigens are the differentiation antigens, which are homogenously expressed by cells of a given tissue type or cell lineage and consequently, by all malignant cells arising therefrom. Given that these antigens are concomitantly expressed in healthy tissues, therapeutic efficacy is generally accompanied by ’on-target’ toxicity. For example, adoptive cell transfer directed against the metastatic melanoma differentiation antigens gp1000 and melanoma-associated antigen recognised by T cells (MART-1) resulted in regression in 30% of patients, though these individuals frequently experienced uveitis and hearing loss due to destruction of melanocytes in the eye and ear [180]. Similarly, targeting carcinoembryonic antigen (CEA) overexpression in metastatic CRC induced regression but also severe inflammatory colitis [181].

### Harnessing NK Cells for Innate Immunotherapy

In recent years, NK cells have emerged as alternative candidates for immunotherapeutic development. Certainly, the MHC-I unrestricted manner of NK cell responses may render this subset a more promising candidate for immunotherapy, as they may overcome the restricted benefit of antigen-specific T-cells in tumors with high mutational diversity. NK-based therapies may prove a new frontier in the treatment of immunologically ’cold’ or refractory tumors, given that the one of the most common mechanisms of immune escape employed by tumor cells is downregulation of MHC-I machinery [182]. Similarly, defects in genes implicated in antigen processing and presentation have recently been identified as key drivers of acquired resistance to immune checkpoint therapies [183]. Additionally, NK cell cytotoxicity may be triggered following engagement of ligands upregulated by transformed cells in response to epithelial-mesenchymal transition, such as MIC-A/B and ULBP1-3 [184]. The latter renders NK cells particularly apt in the eradication of early metastatic cells. Importantly, NK cell receptors are preformed and thus do not require prior sensitisation, clonal expansion and co-stimulatory signalling required for T-cell responsiveness, thus allowing for more rapid cytotoxic responses. Whilst adoptive transfer of HLA-mismatched NK cells induces graft-versus-tumor effects, these cells do not contribute to dose-limiting graft-versus-host disease (GvHD) and may even play a protective role by dampening alloreactive T-cell responses [185,186].

Although no therapies directed specifically at NK cells have been approved in the clinic to date, such promising data suggests that a next wave of therapeutic advances could come from targeting this cell type (Table 1). In phase I/II clinical trials, monoclonal antibodies targeting NK cell inhibitory receptors, such as NKG2A [187] and the KIR family [188], have been shown to bolster NK cell-mediated cytotoxicity. Chimeric antigen receptor (CAR) NK cells directed against CD19 [189,190], CD2 subset-1 (CS-1) [191] and epidermal growth factor receptor (EGFR) [192,193] have also shown efficacy in xenograft models. To improve specificity, Bi-Specific Killer cell Engagers (BiKEs) have been developed which co-target the CD16 low affinity IgG receptor (FcγRIII) and epitopes expressed by malignant cells, such as CD33 [194] and EpCAM [195]. BiKEs have been shown to mediate NK cell cytotoxicity, which is markedly enhanced following the incorporation of a modified human IL-15 crosslinker to generate a tri-specific moity (TriKE; [196]. Yet, these approaches all still rely on tumor cell expression of the selected target and may therefore show limited success in eliminating heterogenous tumor cell populations. Addressing this challenge, CAR T-cells have been engineered to co-express members of the natural cytotoxicity receptor (NCR) family of NK cell activating receptors (including NKp46 [197], NKp44 [198], and NKp30 [199]. These ’hybrid’ CARs avoid the obstacle of MHC-restriction but retain the long-term persistence of adoptively transferred T-cells, endowing cytotoxic T-cells with an NK cell-like pattern of recognition. It is through such innovations, which consider the complexity of tumor cell heterogeneity and acknowledge that immunotherapy may not be a ’one size fits all’ approach, that we may draw the greatest clinical benefit.

## 5. Concluding Remarks

Whether driven by immune cell-intrinsic or tumor-induced factors, it is clear that a vast scope of immunological heterogeneity exists across human cancers. Incorporating our understanding of this heterogeneity into clinical studies may improve our ability to further stratify patients who are candidates for immunotherapy and aid in the design of rational combination therapies directed against heterogeneously expressed targets thereby complementing existing therapeutic strategies, such as those targeting PD-L1. Additionally, further research exploring the influence of TMB on the infiltration and effector functions of non-antigen restricted mediators, specifically NK cells, could inform new therapeutic strategies harnessing the innate immune compartment.

## Figures and Tables

**Table 1:** Completed and currently active clinical trials of NK cell-based immunotherapies.

Approach	Target		Indication	Phase	Clinical Trial ID(s)
Adoptive cell transfer:	Allogenic PBMCs (non-targeted)		Leukemias and lymphomas	Phase I/II	NCT00569283; NCT00799799; NCT00823524; NCT00303667; NCT00187096; NCT00274846; NCT01106950; NCT005626292; NCT01390402; NCT02395822; NCT00586690; NCT00586703; NCT00145626; NCT01386619; NCT00945126; NCT00354172; NCT01313897; NCT01181258
			Solid cancers	Phase I/II	NCT01287104; NCT01212341; NCT01105650; UMIN000013378; NCT01147380; 2005-005125-58
	Autologous PBMCs (non-targeted)		Multiple myeloma	Phase I	NCT02481934
			Advanced digestive cancer	Phase I	UMIN000007527
			Advanced melanoma or kidney cancer	Phase II	NCT00328861
	NK-92 (NK cell line; non-targeted)		Advanced renal cell cancer or melanoma	Phase I	N/A [200]
			End-stage chemotherapy resistant cancer	Phase I	N/A [201]
			Hematologic malignancies		NCT00990717
			Relapsed acute myeloid leukemia	Phase I	NCT00900809
			Stage IIIB or Stage IV Merkel cell carcinoma	Phase II	NCT02465957
Chimeric Antigen Receptors	CD19		Solid and hematological malignancies	Phase I/II	NCT03690310; NCT03679927; NCT03056339; NCT01974479; NCT00995137; NCT02892695
	ROBO1		Solid tumors	Phase I/II	NCT03940820
	BCMA		Relapsed and refractory multiple myeloma	Phase I/II	NCT03940833
	PSMA		Castration-resistant prostate cancer	Phase I	NCT03692663
	NKG2D		Metastatic solid tumors	Phase I	NCT03415100
	Mesothelin		Epithelial ovarian cancer	Not yet recruiting	NCT03692637
	CD33 CAR NK-92		Acute myeloid leukemia	Phase I/II	NCT02944162
	CD7		Lymphoma and leukemia	Phase I/II	NCT02742727
	MUC1		Solid tumors	Phase I/II	NCT02839954
	HER2		Glioblastoma	Phase I/II	NCT03383978
	NKG2D ligands		Solid tumors	Phase I	NCT03415100
Bi- and Tri- specific Killer cell Engagers (BiKe/TriKes)	CD16 × CD33		Myelodysplastic syndromes	Pre-clinical	N/A [194]
	AFM13 (CD30 × CD16A) BiKe		Hodgkin lymphoma	Phase I	NCT01221571
	AFM13 (CD30 × CD16A) BiKe		Relapsed/refractory cutaneous lymphomas	Phase I/II	NCT03192202
	CD16 × IL-15 × CD33 TriKe		AML & high-risk myelodysplastic syndromes	Phase I/II	NCT03214666
Anti-NKG2A	Monalizumab (anti-NKG2A) + cetuximab (anti-NKG2A)		Squamous cell carcinoma of the head and neck	Phase II	NCT02643550
	Monalizumab (anti-NKG2A) + durvalumab (anti-NKG2A)		Advanced or metastatic solid cancers	Phase I/II	
Anti-KIR	KIR3DL2				
		IPH4102	Cutaneous T-cell lymphoma	Phase I	NCT02593045
		IPH4102 +/− gemcitabine +/− oxaliplatin	Advanced T-cell lymphoma	Phase II	NCT03902184
	KIR2DL1-,2-,3-:				
		Lirilumab (IPH2102/BMS-986015)	Smoldering multiple myeloma	Phase II	NCT01222286
		Lirilumab (IPH2102/BMS-986015)	Acute myeloid leukemia	Phase II	NCT01687387
		Lirilumab (IPH2102/BMS-986015) + ipilimumab (anti-PD-1)	Advanced solid tumors	Phase I/II	NCT01714739 & NCT03203876
		Lirilumab (IPH2102/BMS-986015) + ipilimumab (anti-CTLA-4)	NSCLC, Castration Resistant Prostate Cancer, Melanoma	Phase I	NCT01750580
		Lirilumab (IPH2102/BMS-986015) + ipilimumab (anti-CD20)	Chronic lymphocytic leukemia	Phase II	NCT02481297
		1-7F9 (IPH2101)	Multiple myeloma	Phase I	NCT0055296 & NCT00999830
		1-7F9 (IPH2101)	Acute myeloid leukemia	Phase I	NCT01256073

## Abbreviations

Common cell types and acronyms used throughout this manuscript.

CD56^bright^ natural killer (NK) cell	Immunoregulatory subset (~10%) of NK cells producing type I pro-inflammatory cytokines
CD56^dim^ NK cell	Cytotoxic subset (~90%) of NK cells characterized by high production of perforin and granzyme B.
Chimeric antigen receptor (CAR)	Chimeric proteins that fuse an extracellular tumor antigen-targeting domain with a lymphocyte (T- or NK cell)-activating intracellular moiety.
Bi-/Tri-specific killer cell engager (Bi-/TriKE)	Advanced biologicals engineered to express antibody domains capable of binding multiple unique antigens (e.g., 2 antigens /Bi- or 3 antigens/Tri on NK cells and tumor cells to promote NK cell activation and binding to tumor cells)
Killer cell immunoglobulin-like receptor (KIR)	Large family of highly polymorphic NK cell receptors (also expressed in a subset of T-cells) which regulate cytotoxicity by engaging “self” molecules, such as MHC-I.
Major histocompatibility complex class I (MHC-I)	Multi-protein complex expressed by all nucleated cells in mammals. MHC-I presents peptide fragments (derived from self, non-self and neo-antigens) to cytotoxic T-cells.
Natural cytotoxicity receptor (NCR)	Family of type I transmembrane proteins which, when stimulated, trigger NK cell degranulation and cytotoxicity; most tumor-associated NCR ligands are unknown.
Neoantigen-dependent killing	Peptides arising from tumor mutations are presented to T-cells in the context of MHC-I, triggering clonal expansion of cytotoxic T-cells which specifically target tumor cells expressing the cognate neoantigen.
Neoantigen-independent killing	Cytotoxicity which does not require priming by a specific antigen; NK cell cytotoxicity is antigen-independent and therefore not restricted to tumor cells that express the cognate neoantigen.
Microsatellite instability (MSI)	Type of genetic instability arising from defective DNA mismatch repair, resulting in a hypermutated phenotype.
Tumor-infiltrating lymphocyte (TIL)	Lymphocyte which has migrated from the peripheral blood into a solid tumor; This term often refers to tumor-infiltrating cytotoxic CD8+ T-cells.
Tumor mutation burden (TMB)	Number of mutations per coding area of a tumor genome; high TMB is associated with better responses to checkpoint immunotherapy.
